# A systematic review and meta-analysis of the treatment modalities available for children afflicted from cystic fibrosis

**DOI:** 10.1186/s12887-025-06161-y

**Published:** 2025-10-03

**Authors:** Thamer Alshami Marghel Alruwaili, Muhannad Faleh Alanazi, Bashayer Farhan Alruwaili, Hamada K. Fayed, Mahmoud Elsaeed, Talal Difallah Alnazi, Ahmad Aljared, Aasheq M. W. Alanazi, Abdulrahman M. Alanazi, Saleh Dhifallah Alsharari, Amirah Alshammari, Khaled Saad

**Affiliations:** 1 Department of Pediatrics, College of Medicine, Jouf University, Sakaka, 72388 Saudi Arabia; 2 Department of Internal Medicine, Division of Radiology, College of Medicine, Jouf University, Sakaka, Saudi Arabia; 3 Department of Family and Community Medicine, College of Medicine, Jouf University, Sakaka, 72388 Saudi Arabia; 4 Department of Chest Diseases, Faculty of Medicine, Al-Azhar University, Assiut, Egypt; 5 Department of Chest Diseases, Faculty of Medicine, Al-Azhar University, Cairo, Egypt; 6 College of Medicine, Jouf University, Sakaka, 72388 Saudi Arabia; 7 Riyadh Second cluster, MOH, Riyadh, Saudi Arabia; 8 Department of Pediatrics, Faculty of Medicine, Assiut University, Assiut, 71515 Egypt

**Keywords:** Cystic fibrosis, Elexacaftor, Tezacaftor, Ivacaftor, Physiotherapy, Azithromycin, Meta-analysis

## Abstract

**Background:**

This study aimed to evaluate the efficacy of different treatment modalities in children with cystic fibrosis (CF) and determine the superiority of specific treatment modalities.

**Methods:**

A comprehensive literature search was conducted using different search strings across multiple databases, including PubMed, Cochrane Library, EMBASE, WOS, Scopus, CINAHL, PsycINFO, and Google Scholar, up to October 2024. Randomized controlled trials (RCTs), case-control studies and cohort studies were included.

**Results:**

The triple therapy indicated a significant reduction in CF-related complications, with an OR of 0.29 and an RR of 0.54, accompanied by low heterogeneity (I² = 0% for both). Physiotherapy and pulmonary exercises also yielded a beneficial effect, with an OR of 0.24 and an RR of 0.49, without heterogeneity. In contrast, nutritional interventions revealed non-significant outcomes (OR = 6.91 and RR = 2.63), suggesting the need to re-evaluate these strategies. Ivacaftor alone did not achieve statistical significance (OR = 0.34 and RR = 0.58), and the confidence intervals were broad, indicating uncertainty in the effect estimates. Azithromycin exhibited a positive effect on CF management, with an OR of 2.37 and an RR of 1.54. The overall pooled OR across all treatments was 0.71, with an RR not computed due to substantial heterogeneity (I²=93%).

**Conclusion:**

The study underscores the effectiveness of certain treatments, such as triple therapy and physiotherapy exercises, for CF while highlighting the considerable variability in treatment outcomes. Notably, nutritional interventions need to be carefully reassessed. The findings emphasize integrating physiotherapy and targeted pharmacological interventions into standard CF management tailored to individual needs.

**Supplementary Information:**

The online version contains supplementary material available at 10.1186/s12887-025-06161-y.

## Key points

• Genetic testing and treatment advances have recently improved outcomes for people with cystic fibrosis (CF).

• The study highlights the therapeutic advancements brought by *CFTR* modulators, such as Ivacaftor, in improving pulmonary functions and quality of life (QoL) for children with CF, though variability in individual responses persists.

• Evidence supports integrating physiotherapy and pharmacological approaches as essential components of CF management to optimize patient outcomes.

• The findings emphasize integrating physiotherapy and targeted pharmacological interventions into standard CF management tailored to individual needs.

**Supplementary Information:**

The online version contains supplementary material available at 10.1186/s12887-025-06161-y.

## Introduction

Cystic fibrosis (CF) is a complicated hereditary illness that particularly affects the respiratory and digestive systems. Variants in the *CFTR* gene induce aberrant function or the complete lack of the *CFTR* protein, which causes cystic fibrosis [1]. The *CFTR* protein controls chloride ion transport across cell membranes, which is essential for the appropriate distribution of salt and water throughout the body [2]. Due to the faulty *CFTR* protein in CF [3]sticky thick mucus is produced in the lungs and other afflicted organs. This thick mucus blocks airflow, which in turn causes lung infections, inflammation, and eventually permanent lung damage [4]. Additionally, the impaired *CFTR* function affects the function of various other organs, including the liver, pancreas, and reproductive system [5, 6]. The severity of the clinical manifestations of CF varies from person to person. Symptoms frequently encountered include colds, wheezing, shortness of breath, slowed development, low appetite, and salty-tasting skin. Complications from CF can also result in infertility, diabetes, liver disease, and malnutrition [7].

Diagnosing cystic fibrosis involves clinical examination, genetic testing, and specialized diagnostic methods such as sweat chloride and lung function tests. An early diagnosis is required to initiate suitable care measures to maintain lung function and minimize consequences [8]. The *CFTR* gene has around 2,000 identified variants, and these variations may affect CF [9, 10] severity and clinical presentation in various ways. The most common variant, F508del, causes a considerable fraction of instances of CF. However, many other less frequent variants can potentially cause cystic fibrosis [11]. Genetic testing is available to identify CF carriers and to diagnose CF in affected individuals. For families with a CF family history or those living in a high-carrier-frequency community, carrier testing may be used to estimate the likelihood of producing a kid with the disease [9]. Diagnostic testing involves analyzing the *CFTR* gene for variants using DNA sequencing or targeted mutation analysis [11].

Genetic testing and treatment advances have recently improved outcomes for people with CF. Lung function may be improved, and lung infections can be reduced in persons with CF by using gene therapy or *CFTR* modulators, which treat the underlying genetic abnormality in CF [7, 11]. Given the growing number of treatment modalities and the variability in clinical responses among patients with CF, particularly in pediatric populations, there is a growing need to evaluate the current evidence base. Therefore, we conducted this systematic review and meta-analysis to evaluate the efficacy of various treatment modalities—including CFTR modulators, physiotherapy, nutritional support, and antibiotics—in improving clinical outcomes in children with CF. Our aim was to identify those treatments with the strongest supporting evidence and to highlight areas where further research is warranted.

## Materials and methods

### Implementation of the PICO protocol

The PICO strategy employed for the study is as follows-

- Population: Cystic fibrosis in children (under the age of 18).
- Intervention: Modalities for Treating Cystic Fibrosis (e.g., antibiotics, bronchodilators, pancreatic enzymes, chest physiotherapy, exercise, gene therapy, *CFTR* modulators).
- Comparison: Placebo or other treatment modalities.
- Outcome: Improved lung function, decreased incidence and severity of lung infections, improved nutritional status, enhanced quality of life (QoL), and prolonged lifespan.

Using the PICO framework, the research question can be formulated as follows: “In children with cystic fibrosis, what is the effectiveness of various treatment modalities compared to placebo or other treatments in improving lung function, reducing the frequency and severity of lung infections, improving nutritional status, QoL, and overall survival?”

This research question guided the meta-analysis and systematic review by helping to define the inclusion and exclusion criteria for studies, identify relevant outcome measures, and further guide the synthesis and analysis of the data.

### Database search protocol

**Fig. 1:**
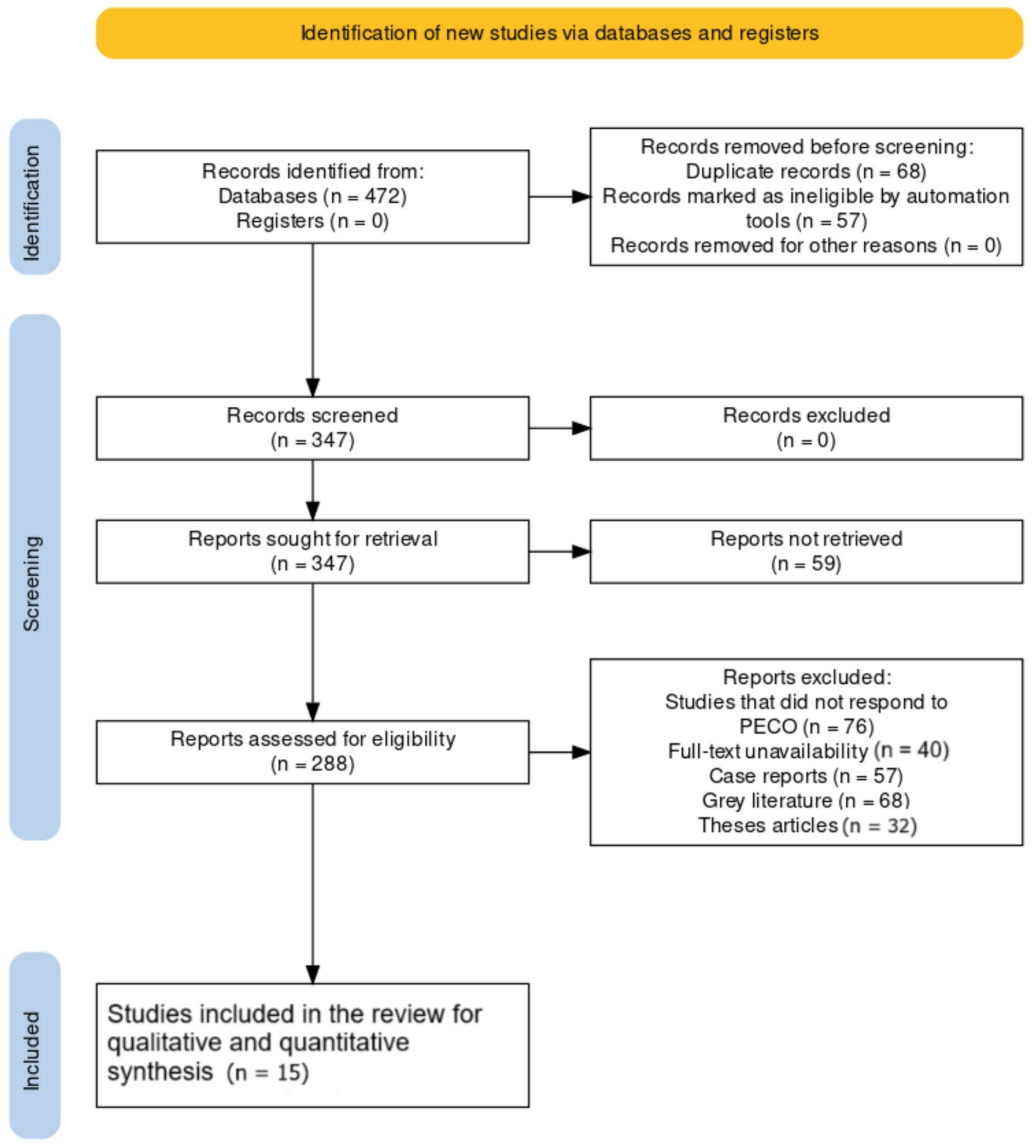
Framework implemented for study selection in this study

The Preferred Reporting Items for Systematic Review and Meta-analysis (PRISMA) protocol [12]illustrated in more detail in Fig. 1, was used to conduct this systematic review. Relevant keywords, citation searches, and reference searches were used to search WOS, EMBASE, PubMed, Cochrane Library, Scopus, PsycINFO, CINAHL, and Google Scholar. The database was searched using the phrases “Antibiotics,” “Cystic fibrosis,” “*CFTR* mutation,” “F508del,” “Pancreatic insufficiency,” and “Phe508del.” Through this systematic review and subsequent meta-analysis, we aimed to assess the wide range of treatment choices available for children with CF and analyse the various modalities in the papers we selected after adopting the required inclusion/exclusion criteria. Table 1 shows the search strategy that was implemented for this review.

**Table 1:** Search strings utilized across the databases

Database	Search string	Boolean operator/MeSH terms used
PubMed	(“Cystic Fibrosis“[MeSH Terms] AND “Child“[MeSH Terms] AND (“Anti-Bacterial Agents“[MeSH Terms] OR “Anti-Inflammatory Agents“[MeSH Terms] OR “Bronchodilator Agents“[MeSH Terms] OR “Mucolytic Agents“[MeSH Terms] OR “Genetic Therapy“[MeSH Terms] OR “Enzyme Replacement Therapy“[MeSH Terms] OR “Diet Therapy“[MeSH Terms] OR “Chest Physical Therapy“[MeSH Terms] OR “Exercise Therapy“[MeSH Terms] OR “CFTR Modulators“[MeSH Terms]))	AND, OR, [MeSH Terms]
EMBASE	(‘cystic fibrosis’/exp AND ‘child’/exp AND (‘antibiotic agent’/exp OR ‘anti-inflammatory agent’/exp OR ‘bronchodilator agent’/exp OR ‘mucolytic agent’/exp OR ‘genetic therapy’/exp OR ‘enzyme replacement therapy’/exp OR ‘diet therapy’/exp OR ‘physical therapy’/exp OR ‘exercise therapy’/exp OR ‘CFTR modulator’/exp))	AND, OR,/exp
Cochrane Library	(“Cystic Fibrosis“[MeSH] AND “Child“[MeSH] AND (“Anti-Bacterial Agents“[MeSH] OR “Anti-Inflammatory Agents“[MeSH] OR “Bronchodilator Agents“[MeSH] OR “Mucolytic Agents“[MeSH] OR “Genetic Therapy“[MeSH] OR “Enzyme Replacement Therapy“[MeSH] OR “Diet Therapy“[MeSH] OR “Chest Physical Therapy“[MeSH] OR “Exercise Therapy“[MeSH] OR “CFTR Modulators“[MeSH]))	AND, OR, [MeSH]
Web of Science	(TS=(“Cystic Fibrosis”) AND TS=(“Child”) AND TS=(“Antibiotics” OR “Anti-Inflammatory Agents” OR “Bronchodilators” OR “Mucolytics” OR “Gene Therapy” OR “Enzyme Therapy” OR “Nutritional Therapies” OR “Physical Therapy” OR “Exercise Therapy” OR “CFTR Modulators”))	AND, OR, TS=
Scopus	(TITLE-ABS-KEY (“cystic fibrosis” AND “child” AND (“antibiotics” OR “anti-inflammatory agents” OR “bronchodilators” OR “mucolytics” OR “gene therapy” OR “enzyme replacement therapy” OR “dietary therapy” OR “physical therapy” OR “exercise therapy” OR “CFTR modulators”)))	AND, OR, TITLE-ABS-KEY
CINAHL	(MH “Cystic Fibrosis+” AND MH “Child+” AND (MH “Antibiotics+” OR MH “Anti-Inflammatory Agents+” OR MH “Bronchodilators+” OR MH “Mucolytics+” OR MH “Genetic Techniques+” OR MH “Enzyme Replacement Therapy+” OR MH “Diet Therapy+” OR MH “Physical Therapy+” OR MH “Exercise+” OR MH “CFTR Modulators+”))	AND, OR, MH
PsycINFO	(DE “Cystic Fibrosis” AND DE “Child” AND (DE “Antibiotics” OR DE “Anti-Inflammatory Agents” OR DE “Bronchodilators” OR DE “Mucolytics” OR DE “Genetic Procedures” OR DE “Enzyme Therapy” OR DE “Nutritional Therapies” OR DE “Physical Therapy” OR DE “Exercise” OR DE “CFTR Modulators”))	AND, OR, DE
Google Scholar	“Cystic fibrosis” AND “child” AND (“antibiotics” OR “anti-inflammatory” OR “bronchodilators” OR “mucolytics” OR “gene therapy” OR “enzyme therapy” OR “nutritional therapy” OR “physical therapy” OR “exercise therapy” OR “CFTR modulators”)	-

### Criterion for selection

Table 2 shows the inclusion and exclusion criteria devised for the review.

**Table 2:** Inclusion and exclusion criteria devised for this review

Criteria	Inclusion	Exclusion
Language	Studies published in English	Studies published in languages other than English
Population	Children (< 18 years old) with a confirmed diagnosis of cystic fibrosis	Studies not involving children with cystic fibrosis
Intervention	Various modalities for treating cystic fibrosis (e.g., antibiotics, bronchodilators, etc.)	Studies not evaluating treatment modalities for cystic fibrosis
Outcomes	Reports on treatment efficacy, safety, and/or quality of life (QoL)	Studies not reporting on treatment efficacy, safety, and/or QoL
Study Design	Randomized controlled trials (RCTs), cohort studies, case-control studies, and cross-sectional studies	Case reports, letters, editorials, or review articles
Comparison	Placebo or other treatment modalities	-
Reported outcomes	Improved lung function, reduced severity and incidence of lung infections, improved nutritional status, enhanced QoL.	-

### Reviewer protocol

The process of conducting this review required careful planning to ensure the accuracy and reliability of the results. A critical aspect of this process was the implementation of a data selection protocol using multiple reviewers, which aimed to reduce bias and increase the validity of the findings. The first stage of the process was to locate appropriate research. At least two reviewers separately examined titles and abstracts for relevance to predetermined inclusion and exclusion criteria. Then, each reviewer separately performed a full-text evaluation of the papers that had fulfilled the inclusion criteria. Any discrepancies or conflicts were explored and resolved by reaching a consensus or bringing in a third reviewer.

Separately from each other, data was extracted from the entire text by two reviewers, using a standardized form that included relevant study characteristics. Each study was assessed for quality using a standardized tool and independently by at least two reviewers. When disagreements arose, they were addressed and resolved either by reaching a consensus or bringing in a third reviewer. Finally, a meta-analysis combined the data, involving the computation of effect sizes and pooled estimates for every treatment modality. Additionally, a sensitivity analysis was performed to evaluate the reliability of the meta-analysis findings. The reviewers analysed the data and produced the final results using statistical software.

### Statistical protocol

Using the RevMan 5 program, forest plots were created for the ratio of risk, risk difference, and odds ratio as part of the statistical analysis methodology. When comparing each treatment modality to the control group, the odds ratio forest plot showed the probability of occurrence with a 95% confidence interval. Compared to the control group, the risk ratio estimates and 95% confidence intervals for each treatment method were shown in the risk ratio forest plot. Compared to the control group, the risk difference forest plot showed the risk difference estimate and 95% confidence intervals for each treatment method. The random effects model was employed because of the anticipated heterogeneity across the included papers since the treatment modalities to be investigated would be of different types.

The reviewers created the forest plots using Revman 5 software and utilized statistical software to determine the effect sizes and pooled estimates for each treatment modality. Each forest plot showed the findings from every study, together with a pooled estimate and matching confidence interval. The panel of reviewers also conducted a sensitivity analysis, which involved assessing the degree to which different study features affected the final aggregated numbers to ascertain how stable the findings were.

By creating the forest plots using the Revman 5 software, the reviewers were able to visually represent the data and present a thorough summary of the treatment options available for children with cystic fibrosis.

### Bias evaluation protocol

**Fig. 2:**
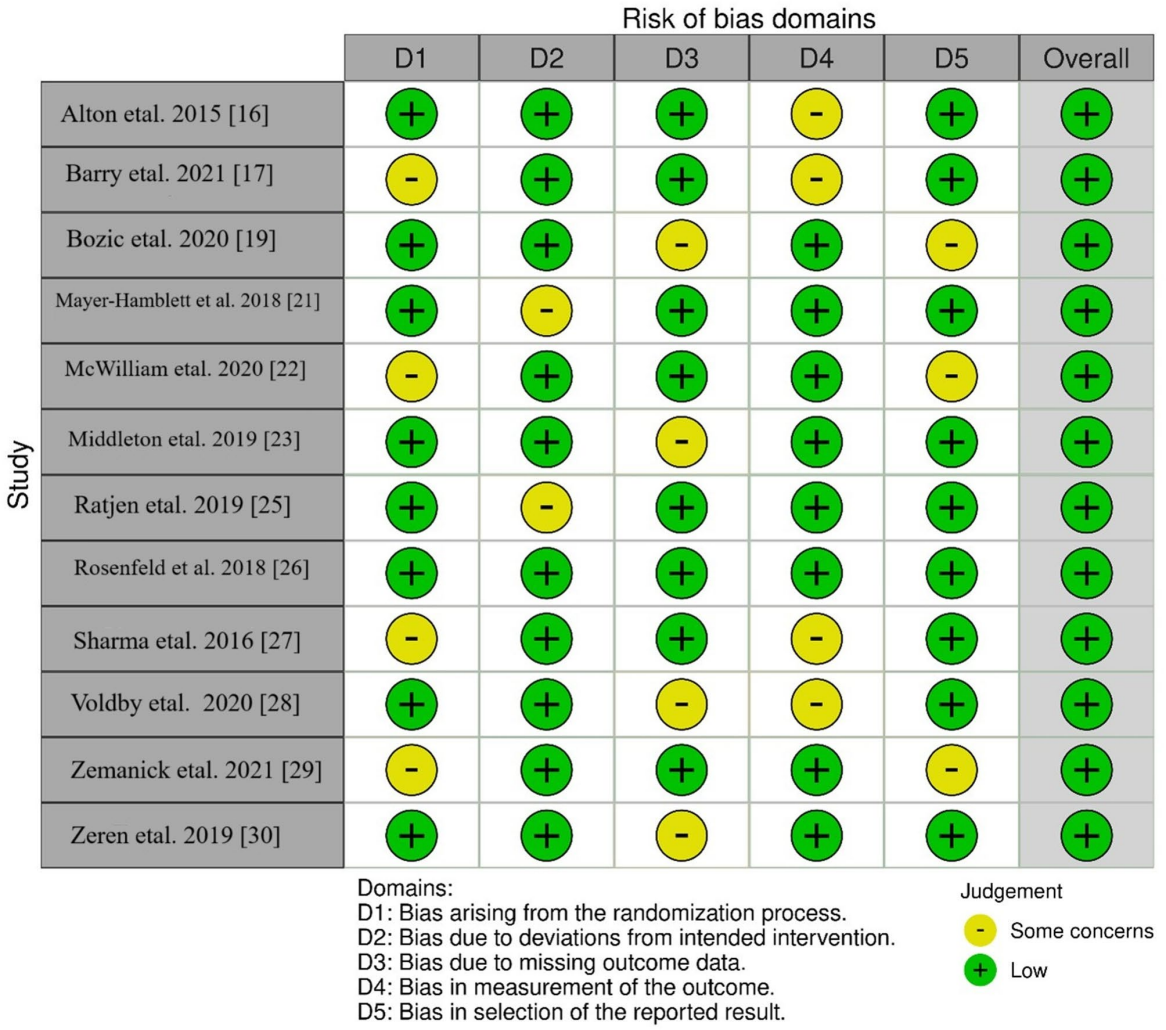
Bias assessment in the RCTs included in the review using RoB 2.0

**Fig. 3:**
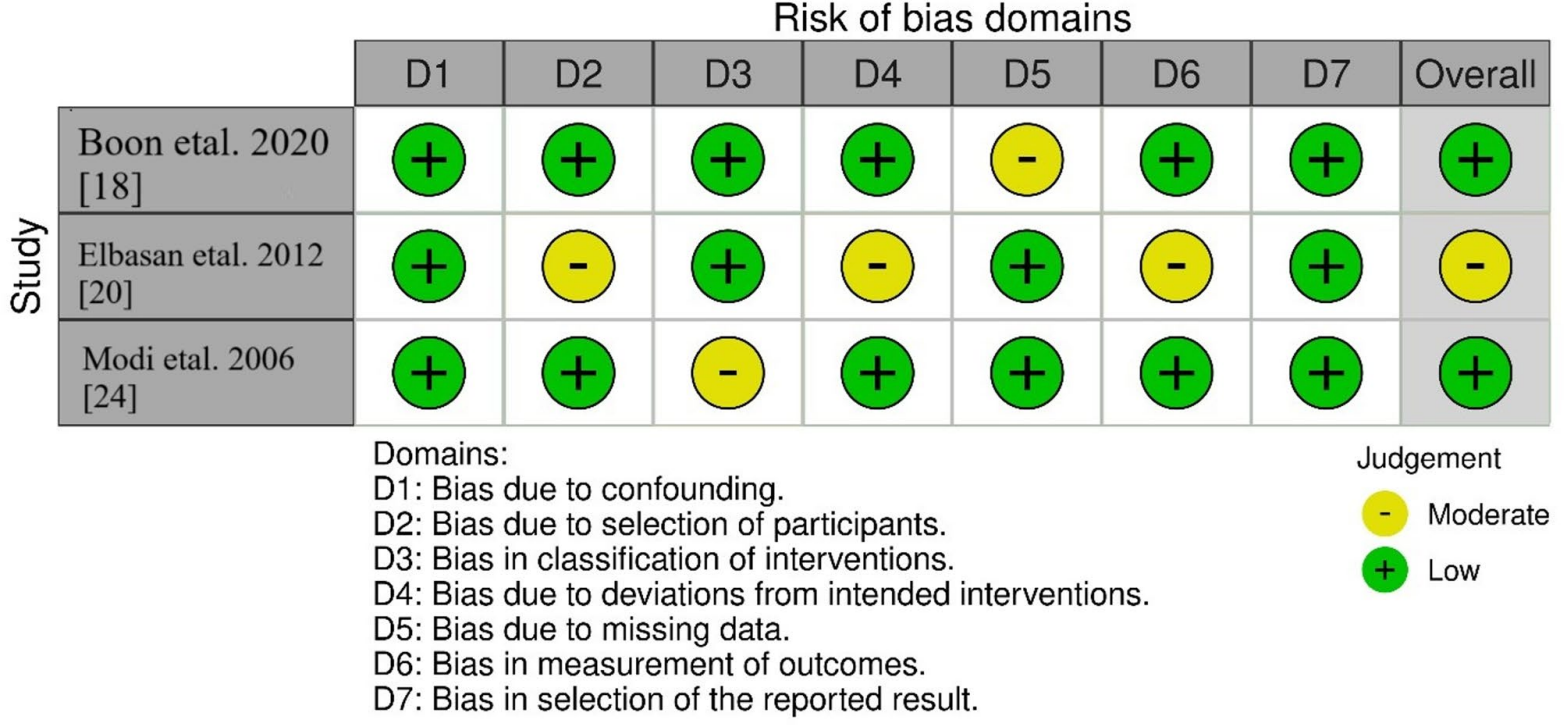
Bias assessment in the observational studies included in the review using ROBINS-I

Evaluation of the potential for bias in this meta-analysis and systematic review of treatments for children with CF were conducted using the Cochrane risk-of-bias tool for randomized trials (RoB 2.0) tool [13] and Risk of Bias in Non-randomized Studies - of Interventions (ROBINS-I) tool [14]. Figures 2 and 3 show the results of this assessment across various domains.

### Certainty assessment

The GRADE framework [15] was executed to ascertain the robustness of the clinical directives and the integrity of the evidence underpinning the systematic review, as delineated in Table 3. The GRADE system incorporates a multifaceted analytical paradigm engaging several domains to determine the aggregate certainty of the evidence base. These domains encompass an evaluation of potential biases, the consistency of the evidence, the directness of the evidence, the precision of the effect estimates, and other considerations, such as the presence of publication bias, all in conjunction with the application of the revised RoB 2.0 ^13^ and the ROBINS-I instrument [14].

**Table 3:** GRADE assessment observations of the selected studies

Study design	Number of studies	Observed common finding	Risk of Bias	Inconsistency	Indirectness	Imprecision	Others	Certainty
RCT	12	Significant improvements in lung function and quality of life measures in various interventions. Some interventions showed no significant benefit.	Low to Moderate	Low	Low	Low	None	High
Observational	3	Mixed findings regarding the benefits of interventions on lung function and quality of life measures.	Low	Low	Moderate	Low	None	Moderate

## Results

### Article selection schematics

The study selection process for the systematic review began with identifying new studies by extensively searching various databases and registers. Initially, a total of 472 records were identified from databases, with no additional records found in registers. Prior to screening, 68 duplicate records were removed, followed by the elimination of 57 records deemed ineligible. No records were removed for other unspecified reasons. The remaining 347 records were then screened. All of these screened records were subsequently sought for retrieval. However, no records were excluded at this stage. During the retrieval process, 59 reports were not retrieved for further assessment. The next phase assessed 288 reports for eligibility against the predefined inclusion criteria based on PECO. During this assessment, 76 studies that did not respond to the PECO criteria were excluded, along with 40 due to full-text unavailability. Additionally, certain types of literature were deemed inappropriate for inclusion; this resulted in the exclusion of 57 case reports, 68 pieces of grey literature, and 32 theses articles. After this rigorous selection process, only 15 [16–30] studies met all the necessary criteria and were included in the systematic review Table 4.

**Table 4:** Overviews of the findings from the studies that were selected

Author	Year of Study	Country	Sample size	Mean age and male: female ratio	Study design	Study groups
Alton et al. [16]	2015	United Kingdom	40 patients	< 18 years; 54:46	RCT	62 individuals in one group and 78 in the other
Barry et al. [17]	2021	United Kingdom	258 patients	12–18 years; 50.8:49.2	RCT	132 individuals in one group and 126 in the other
Boon et al. [18]	2020	Multicenter (Lisbon, Madrid, Milan, Leuven, Rotterdam, Valencia)	171 patients	2–18 years; 51.46:48.54	Observational	171 patients divided into two equal groups.
Bozic et al. [19]	2020	USA	58 patients	2–10 years; 58.4:41.6	RCT	60 patients divided into two equal groups
Elbasan et al. [20]	2012	Turkey	16 patients	5–13 years; NA	Observational	Sixteen patients were divided into three unequal groups.
Mayer-Hamblett et al. [21]	2019	USA	221 patients	7 years; 55.9:44.1	RCT	Two treatment groups (110 individuals in one, 111 in the other)
McWilliam et al. [22]	2020	United Kingdom	50 patients	6–18 years; 6:44	RCT	Two treatment groups (20 individuals in one, 24 in the other)
Middleton et al. [23]	2019	United Kingdom	116 patients	12–18 years; 52:48	RCT	Two treatment groups (200 individuals in one, 203 in the other)
Modi et al. [24]	2006	USA	37 patients	6–13 years; NA	Observational	Undefined
Ratjen et al. [25]	2019	Multicenter (Canada and USA)	150 patients	3–6 years; NA	RCT	Two treatment groups (76 individuals in one and 74 in the other).
Rosenfeld et al. [26]	2018	USA	19 patients	15.2 months; 57.9:42.1	RCT	Seven children were enrolled in part A and 19 in part B
Sharma et al. [27]	2016	India	40 patients	5–15 years; 60:40	RCT	Two treatment groups (20 individuals in one and 20 in the other)
Voldby et al. [28]	2020	Denmark	29 patients	5–18 years; 51:49	RCT	Two equal treatment groups with randomized allocation
Zemanick et al. [29]	2021	Multicenter (Canada, Australia, Ireland, UK and USA)	66 patients	6–11 years; 40.9:59.1	RCT	Two treatment groups (36 individuals in one and 30 in the other)
Zeren et al. [30]	2019	United Kingdom	36 patients	8–18 years; 44:56	RCT	36 children were randomly allocated into two equal groups

### GRADE assessment results

The review encompassed 12 RCTs, which observed significant improvements in lung function and QoL measures across various interventions, although some did not show significant benefits. The RCTs were generally characterized by a low to moderate risk of bias, indicating a reliable level of data quality. Regarding the GRADE criteria, there was low inconsistency and low indirectness among the RCTs, suggesting that the results were consistent across different studies and directly applicable to the patient population in question. The RCTs also demonstrated low imprecision, meaning the data were sufficiently precise to support a robust conclusion. The certainty of the evidence from the RCTs was rated as high, reflecting strong confidence in the observed effects of the interventions.

In contrast, the review included three observational studies, which presented mixed findings regarding the impact of interventions on lung function and QoL measures. These studies were found to have a low risk of bias and low inconsistency, indicating that the results were reasonably reliable and consistent. However, the observational studies had moderate indirectness, suggesting some uncertainty about the applicability of the findings to the broader CF patient population. Imprecision was rated as low, which means that the data were precise enough to conclude, albeit with some reservations due to the nature of observational research. No other considerations were noted that could affect the quality of the evidence. The certainty of the evidence from the observational studies was considered moderate, indicating a moderate level of confidence in the results, which is typical for observational research compared to RCTs.

### Demographic parameters

The studies, predominantly conducted in the United Kingdom and the United States, also incorporated multicentre trials spanning Europe, Canada, India, and Denmark [16–19, 22, 23, 25, 29, 30]. Sample sizes varied significantly, ranging from 16 to 258 patients, indicative of both focused studies and extensive clinical trials [17, 20]. The youngest cohort of patients averaged 15.2 months, and the oldest was observed to be up to 18 years [26]. Gender ratios across studies were near equivalent or not reported [16–19, 21–23, 27, 28, 30].

RCTs were the predominant study design [16, 17, 19, 21–23, 25, 27–30]the rest being observational studies [18, 20, 24].

### Parameters assessed

Table 5 shows the associated inferences and assessments that were drawn from the included papers. Alton et al. [16] investigated the relative change in the percentage of predicted FEV1 (Forced Expiratory Volume in 1 s). Barry et al. [17] focused on the absolute change in the percentage of predicted FEV1, in addition to measuring sweat chloride concentration and the respiratory domain score from the Cystic Fibrosis Questionnaire-Revised (CFQ-R). The study by Boon et al. [18] assessed the gastrointestinal quality of life (GI QoL) using the CF-PedsQL-GI, a pediatric disease-specific tool designed to measure the health-related QoL in children with cystic fibrosis, focusing particularly on gastrointestinal symptoms. Bozic et al. [19] measured weight-for-age z-scores, other anthropometrics, serum and fecal inflammatory markers, and other clinical outcomes.

**Table 5:** Studies included in the review and their associated inferences

Author	Parameters assessed	Investigated treatment modality	Statistical assessments observed	Overall inference drawn
Alton et al. [16]	- Relative change in % predicted FEV1	- Nebulized pGM169/GL67A gene–liposome complex vs. 0.9% saline (placebo)	- Significant treatment effect at 12 months in pGM169/GL67A group versus placebo (3.7%, 95% CI 0.1–7.3; *p* = 0.046)	- Monthly application of pGM169/GL67A was associated with a significant, albeit modest, benefit in FEV1 compared with placebo at 1 year, indicating stabilization of lung function. No significant difference in treatment-attributable adverse events between groups.
Barry et al. [17]	- Absolute change in % predicted FEV1 - Sweat chloride concentration - Cystic Fibrosis Questionnaire-Revised respiratory domain score	- Elexacaftor–tezacaftor–ivacaftor vs. active control	- Higher FEV1 by 3.7% points and lower sweat chloride by 22.3 mmol per liter relative to baseline with elexacaftor–tezacaftor–ivacaftor (*p* < 0.001). Higher FEV1 by 3.5% points and lower sweat chloride by 23.1 mmol per liter relative to active control (*p* < 0.001). - Improved respiratory domain score by 10.3 points with elexacaftor–tezacaftor–ivacaftor compared to 1.6 with active control.	Elexacaftor, tezacaftor, and ivacaftor showed significant improvement in lung function, a reduction in sweat chloride concentration, and better quality of life outcomes than the active control. Adverse events were similar between the groups, with few leading to treatment discontinuation.
Boon et al. [18]	- Gastrointestinal quality of life (GI QOL) via CF-PedsQL-GI	- Mobile app for self-management of PERT in CF patients	- Significant improvement from M0 to M6 in CF-PedsQL-GI scores (*p* < 0.0001). Greater improvement is associated with lower baseline scores (*p* < 0.001).	- The MyCyFAPP mobile app may improve GI QOL in children with CF and support better self-management of PERT, particularly in those with significant GI symptoms.
Bozic et al. [19]	- Weight-for-age z-scores - Anthropometrics - Serum and fecal inflammatory markers - Other clinical outcomes	- Oral glutathione vs. placebo in pancreatic insufficient CF patients	- No significant differences in primary or secondary endpoints between glutathione and placebo groups (weight-for-age z-score *p* = 0.25; weight change *p* = 0.35; BMI change *p* = 0.69).	- Oral glutathione supplementation did not improve growth or inflammatory markers compared to placebo but was safe and well tolerated in children with CF.
Elbasan et al. [20]	- Physical fitness parameters: cardiovascular endurance, muscle endurance, strength, flexibility, power, and agility	- Chest physiotherapy and aerobic exercise training	- Positive progressions in all assessed fitness parameters except for the 20 m shuttle run and 10 stair climbing tests (*p* < 0.05). No significant change in sit and reach and forward bending tests (*p* > 0.05).	- Combined chest physiotherapy and aerobic exercise training can improve physical fitness, thoracic mobility, and muscle endurance in young CF patients, with no significant impact on agility or flexibility.
Mayer-Hamblett et al. [21]	- Time to pulmonary exacerbation requiring antibiotics - Time to *Pseudomonas aeruginosa* recurrence - Clinical and safety outcomes - Weight changes	- Addition of azithromycin to TIS for children with early *Pseudomonas aeruginosa*	− 44% reduction in risk of pulmonary exacerbation with azithromycin vs. placebo (hazard ratio, 0.56; *p* = 0.004) - Increase in weight by 1.27 kg with azithromycin (*p* = 0.046) - No significant differences in microbiological or other clinical or safety endpoints	- Azithromycin, added to TIS, significantly reduced the risk of pulmonary exacerbation and increased weight without impacting microbiological outcomes in children with early Pa.
McWilliam et al. [22]	- Change in urinary KIM-1 levels - Safety and adverse events	- Oral rosuvastatin during treatment with intravenous tobramycin	- No significant difference in KIM-1 levels between rosuvastatin and control groups (mean treatment difference 1.08, *p* = 0.48) - Low level of nephrotoxicity overall − 12 mild adverse reactions in rosuvastatin group, one serious adverse event in each group	- Rosuvastatin did not demonstrate a protective effect against aminoglycoside-induced nephrotoxicity, but overall nephrotoxicity was lower than expected, leaving the hypothesis inconclusive.
Middleton et al. [23]	- Change in percentage of predicted FEV1 - Rate of pulmonary exacerbations - Respiratory domain score on the CF Questionnaire-Revised - Change in sweat chloride concentration - Safety and side-effect profile	- Elexacaftor–tezacaftor–ivacaftor for patients with Phe508del–minimal function genotypes	- Significant improvement in FEV1 by 13.8 points at 4 weeks and 14.3 points through 24 weeks with the treatment (*p* < 0.001) − 63% lower rate of pulmonary exacerbations (*p* < 0.001) − 20.2 points higher respiratory domain score (*p* < 0.001) − 41.8 mmol/L lower sweat chloride concentration (*p* < 0.001) - Generally safe with an acceptable side-effect profile	- Elexacaftor–tezacaftor–ivacaftor significantly improved lung function and quality of life and reduced sweat chloride levels and pulmonary exacerbations, with an acceptable safety profile in CF patients with Phe508del–minimal function genotypes.
Modi et al. [24]	- Rates of adherence using parent and child self-reports, diary data, pharmacy refill history, and electronic monitors	- Standard CF treatment components	- Rates of overall adherence were below 50% using objective measures. - Variability in adherence by treatment component: enzyme medications (27–46%), airway clearance (64–74%), nebulized medications (48–82%), specific nebulized meds (dornase alpha 57–90%, inhaled tobramycin 36–85%), vitamins (22–94%), enzymes (27–90%). - Self-reported adherence was approximately 80%.	Generally, children’s adherence to the CF treatment regimen is poor, with objective measures indicating less than 50% adherence, but self-reports suggest higher rates.
Ratjen et al. [25]	- Effect of inhaled hypertonic saline on lung clearance index (LCI2·5), serious adverse events	- Inhaled 7% hypertonic saline vs. 0·9% isotonic saline nebulized twice daily for 48 weeks	- Significant improvement in LCI2·5 with hypertonic saline (mean treatment effect − 0·63 LCI2·5 units, 95% CI − 1·10 to − 0·15; *p* = 0.010). - Serious adverse events were few and not treatment-related.	- Inhaled hypertonic saline significantly improved lung ventilation inhomogeneity in young children with CF without a related increase in serious adverse events.
Rosenfeld et al. [26]	- Primary endpoints: PK (part A) and safety (parts A and B) - Secondary endpoints: PK and absolute change from baseline at week 24 in sweat chloride - Tertiary endpoints: Absolute change from baseline at week 24 in growth parameters and markers of pancreatic function	- Oral ivacaftor 50 mg (weight 7 to < 14 kg) or 75 mg (weight ≥ 14 to < 25 kg) every 12 h	- Adverse events were mostly typical for CF children; cough was the most common - Two children experienced serious AEs; one possibly related to ivacaftor - Five children had transaminase elevations > 3 × ULN - Mean absolute change from baseline in sweat chloride was − 73·5 (17·5) mmol/L - Growth parameters maintained; improvements in fecal elastase-1 and IRT; reduction in serum lipase and amylase	- Ivacaftor was generally safe and well tolerated in children 12 to < 24 months with a CFTR gating variant, showing substantial improvements in sweat chloride and maintenance of growth measures. - Early initiation of ivacaftor could potentially preserve pancreatic function.
Sharma et al. [27]	- Need for antibiotics - Pulmonary function tests (PFTs) - Serum zinc levels at baseline and at 12 months	− 30-mg zinc tablets vs. placebo tablets daily for 12 months	- No significant reduction in the need for antibiotics: zinc group 42 (14–97) days, placebo group 38 (15–70) days (*p* = 0.79) - No significant differences in PFTs: percent-of-predicted FEV1 or change in FEV1 values at 12 months (*p* = 0.44) - Similar isolation rates of *Pseudomonas* in respiratory specimens for both groups	- Zinc supplementation did not significantly reduce the need for antibiotics or improve PFTs compared to placebo in children aged 5–15 years with CF.
Voldby et al. [28]	- Lung clearance index (LCI) - z-scores for FEV1 (zFEV1) and BMI (zBMI) - CF Questionnaire-revised (CFQ-R) respiratory symptom score - Findings from chest computed tomography and bronchoalveolar lavage (BAL)	- BAL performed if LCI increased > 1 unit from baseline, followed by antimicrobial treatment if pathogens were present	- No significant difference in the slope for LCI between the groups - No significant difference in zFEV1, zBMI, CFQ-R scores, or chest CT findings between the groups	- LCI-triggered BAL and subsequent antimicrobial treatment did not improve clinical outcomes in closely monitored school-age children with CF.
Zemanick et al. [29]	- Safety and tolerability - Pharmacokinetics - Improvement in percentage predicted FEV1 - CFQ-R respiratory domain score - Lung clearance index2.5 - Sweat chloride concentration - BMI-for-age z-score	- ELX/TEZ/IVA at age-adjusted dosages for 24 weeks	- Safety profile consistent with older populations - Improvements in FEV1, CFQ-R, lung clearance index2.5, sweat chloride, and BMI z-score	- ELX/TEZ/IVA is safe and efficacious in children aged 6–11 with at least one F508del-CFTR allele, supporting its use in this demographic.
Zeren et al. [30]	- Dynamic and static postural stability - Spirometry - Respiratory muscle strength − 6-min walk distance (6MWD)	- Comprehensive chest physiotherapy (PT) vs. inspiratory muscle training (IMT) alongside PT	- No significant differences between groups in LOS score, FVC, FEV1, peak expiratory flow, MEP, or 6MWD - Greater improvement in MIP in the PT + IMT group	- Comprehensive chest PT effectively improves postural stability, spirometry, respiratory muscle strength, and 6MWD; additional IMT provided no further benefits except for MIP.

Elbasan et al. [20] assessed various physical fitness parameters, including cardiovascular endurance, muscle endurance, strength, flexibility, power, and agility. Mayer-Hamblett et al. [21] measured the time to pulmonary exacerbation requiring antibiotics and the time to *Pseudomonas aeruginosa* recurrence. They also looked at clinical and safety outcomes and weight changes to understand the progression of the disease and the efficacy of treatments. McWilliam et al. [22] investigated changes in urinary KIM-1 levels, a biomarker that may indicate kidney injury, alongside safety and adverse events associated with the treatment of cystic fibrosis. Middleton et al. [23] concentrated on changes in the percentage of predicted FEV1 and the rate of pulmonary exacerbations. They also measured the respiratory domain score on the CF Questionnaire-Revised and changes in sweat chloride concentration, in addition to evaluating the safety and side-effect profile of the treatments used. Modi et al. [24] focused on rates of adherence using various methods such as parent and child self-reports, diary data, pharmacy refill history, and electronic monitors.

Ratjen et al. [25] studied the effect of inhaled hypertonic saline on lung clearance index (LCI2·5) and recorded serious adverse events. Rosenfeld et al. [26] had primary endpoints that included pharmacokinetics (PK) and safety, while secondary endpoints were PK and absolute change from baseline at week 24 in sweat chloride concentration. The tertiary endpoints were the absolute change from baseline at week 24 in growth parameters and markers of pancreatic function, providing a broad overview of the treatment’s effects. Sharma et al. [27] looked into the need for antibiotics, pulmonary function tests (PFTs), and serum zinc levels at baseline and 12 months.

Voldby et al. [28] assessed the lung clearance index (LCI), z-scores for FEV1 and BMI, the CFQ-R respiratory symptom score, and findings from chest computed tomography and bronchoalveolar lavage (BAL). Zemanick et al. [29] reported on the safety and tolerability, pharmacokinetics, percent improvement predicted FEV1, CFQ-R respiratory domain score, lung clearance index 2.5, sweat chloride concentration, and BMI-for-age z-score. Zeren et al. [30] investigated the effects of interventions on dynamic and static postural stability, spirometry, respiratory muscle strength, and the 6-minute walk distance (6MWD).

### Modalities assessed

Alton et al. [16] conducted a study where they compared a nebulized pGM169/GL67A gene–liposome complex against 0.9% saline, which served as the placebo. Barry et al. [17] investigated the impact of the drug combination elexacaftor–tezacaftor–ivacaftor compared to active control. Boon et al. [18] examined the utility of a mobile app designed for the self-management of pancreatic enzyme replacement therapy (PERT) in cystic fibrosis patients. Bozic et al. [19] explored the effects of oral glutathione as opposed to placebo in patients with cystic fibrosis who were pancreatic insufficient. Glutathione was studied for its potential antioxidant effects in this patient population. Elbasan et al. [20] evaluated the benefits of chest physiotherapy combined with aerobic exercise training. This intervention aimed to improve the physical health and respiratory function of individuals with cystic fibrosis. Mayer-Hamblett et al. [21] researched the addition of azithromycin to tobramycin inhalation solution (TIS) for children with early *Pseudomonas aeruginosa* infection. Their work aimed to discover whether this combination therapy could provide superior outcomes compared to standard treatment. McWilliam et al. [22] investigated the effects of oral rosuvastatin administered during treatment with intravenous tobramycin. The study aimed to assess whether adding a statin could have beneficial effects during antibiotic treatment for cystic fibrosis.

Middleton et al. [23] studied using elexacaftor–tezacaftor–ivacaftor in patients with Phe508del–minimal function genotypes. This research was part of the broader effort to tailor cystic fibrosis therapies based on specific genetic profiles. Modi et al. [24] focused on the components of standard cystic fibrosis treatment. Their study was likely to evaluate the effectiveness and adherence to the established treatment protocols for cystic fibrosis. Ratjen et al. [25] investigated the effects of inhaled 7% hypertonic saline versus 0.9% isotonic saline nebulized twice daily for 48 weeks. The study was designed to assess the efficacy of hypertonic saline in improving lung function in cystic fibrosis patients. Rosenfeld et al. [26] administered oral ivacaftor at dosages of 50 mg for patients weighing 7 to less than 14 kg and 75 mg for those weighing at least 14 to less than 25 kg every 12 h. Based on weight categories, the study aimed to understand the pharmacokinetics and safety of ivacaftor in young cystic fibrosis patients.

Sharma et al. [27] compared the effects of 30-mg zinc tablets taken daily for 12 months to placebo tablets. This study intended to determine the role of zinc supplementation in treating cystic fibrosis. Voldby et al. [28] performed bronchoalveolar lavage (BAL) if the lung clearance index (LCI) increased by more than 1 unit from the baseline. This was followed by antimicrobial treatment if pathogens were present, aiming to provide targeted treatment based on lung function and infection status. Zemanick et al. [29] conducted a trial with ELX/TEZ/IVA at age-adjusted dosages for 24 weeks, likely investigating the safety, pharmacokinetics, and efficacy of this drug combination in a pediatric population with cystic fibrosis. Zeren et al. [30] compared comprehensive chest physiotherapy (PT) versus inspiratory muscle training (IMT) alongside PT. This study aimed to determine the effectiveness of different physiotherapy interventions in improving respiratory muscle function in cystic fibrosis patients.

### Inferences drawn

Alton et al. [16] observed a significant treatment effect at 12 months in the group receiving the pGM169/GL67A gene–liposome complex compared to the placebo group. The improvement was quantified at 3.7% with a 95% confidence interval of 0.1–7.3, and the result was statistically significant with a p-value of 0.046. Barry et al. [17] reported that patients treated with elexacaftor–tezacaftor–ivacaftor experienced a higher FEV1 by 3.7% points and a lower sweat chloride by 22.3 mmol per Liter relative to baseline. These results were highly statistically significant (*p* < 0.001). Additionally, there was an improved respiratory domain score by 10.3 points with elexacaftor–tezacaftor–ivacaftor compared to 1.6 with the active control. Boon et al. [18] found a significant improvement from M0 to M6 in CF-PedsQL-GI scores, with a p-value of less than 0.0001. The study also found that a greater improvement was associated with lower baseline scores, which was statistically significant (*p* < 0.001). Bozic et al. [19] determined no significant differences in primary or secondary endpoints between the oral glutathione and placebo groups. Measurements such as the weight-for-age z-score, weight change, and BMI change did not show statistical significance, with p-values of 0.25, 0.35, and 0.69, respectively. Elbasan et al. [20] reported positive progressions in all assessed fitness parameters except for the 20 m shuttle run and 10 stairs climbing tests, which showed statistical significance (*p* < 0.05). However, there was no significant change in sit, reach, and forward bending tests (*p* > 0.05). Mayer-Hamblett et al. [21] found a 44% reduction in the risk of pulmonary exacerbation with azithromycin compared to placebo, with a hazard ratio of 0.56 and a p-value of 0.004. The study also reported an increase in weight by 1.27 kg with azithromycin, which was statistically significant (*p* = 0.046). However, there were no significant differences in microbiological or other clinical or safety endpoints.

McWilliam et al. [22] reported no significant difference in KIM-1 levels between the rosuvastatin and control groups, with a mean treatment difference of 1.08 and a p-value of 0.48. The study noted a low level of nephrotoxicity overall, with 12 mild adverse reactions in the rosuvastatin group and one serious adverse event in each group. Middleton et al. [23] documented significant improvements in FEV1 by 13.8 points at 4 weeks and 14.3 points through 24 weeks with the treatment (*p* < 0.001). There was also a 63% lower rate of pulmonary exacerbations (*p* < 0.001), a 20.2 points higher respiratory domain score (*P* < 0.001), and a 41.8 mmol/L lower sweat chloride concentration (*p* < 0.001). The study highlighted that the treatment was generally safe with an acceptable side-effect profile. Modi et al. [24] found that the overall adherence rates to Cystic fibrosis treatment components were below 50% using objective measures. There was variability in adherence by treatment component, with specific nebulized medications like dornase alpha and inhaled tobramycin ranging from 57 to 90% and 36–85%, respectively. Self-reported adherence was approximately 80%. Ratjen et al. [25] observed a significant improvement in LCI 2·5 with hypertonic saline, with a mean treatment effect of − 0·63 LCI 2·5 units and a 95% confidence interval of − 1·10 to − 0·15; the p-value was 0.010. The study noted that serious adverse events were few and not treatment-related and that adverse events were mostly typical for CF children, with cough being the most common. Rosenfeld et al. [26] noted a mean absolute change from baseline in sweat chloride of − 73·5 (17·5) mmol/L. The study also reported that growth parameters were maintained, with improved fecal elastase-1 and IRT and reduced serum lipase and amylase.

Sharma et al. [27] found no significant reduction in the need for antibiotics between the zinc and placebo groups, with days of antibiotic use being 42 for the zinc group and 38 for the placebo group (*p* = 0.79). There were no significant differences in PFTs or percent-of-predicted FEV1 or change in FEV1 values at 12 months. Voldby et al. [28] conducted a study that found no significant differences in the isolation rates of *Pseudomonas* in respiratory specimens between the zinc and placebo groups. Additionally, no significant differences were observed in the slope for lung clearance index (LCI) between the groups. Zemanick et al. [29] reported no significant difference between the study groups in FEV1 z-scores, BMI z-scores, CFQ-R scores, or chest CT findings. However, the safety profile of the treatments was consistent with those seen in older populations, and improvements were noted in FEV1, CFQ-R, lung clearance index 2.5, sweat chloride, and BMI z-score. Zeren et al. [30] investigated the effects of adding inspiratory muscle training (IMT) to physical therapy (PT) and found a greater improvement in maximal inspiratory pressure (MIP) in the PT + IMT group. However, there were no significant differences between groups regarding lung function scores, FEV1, peak expiratory flow, maximal expiratory pressure (MEP), or the 6-minute walk distance (6MWD).

### Statistical observations

**Fig. 4:**
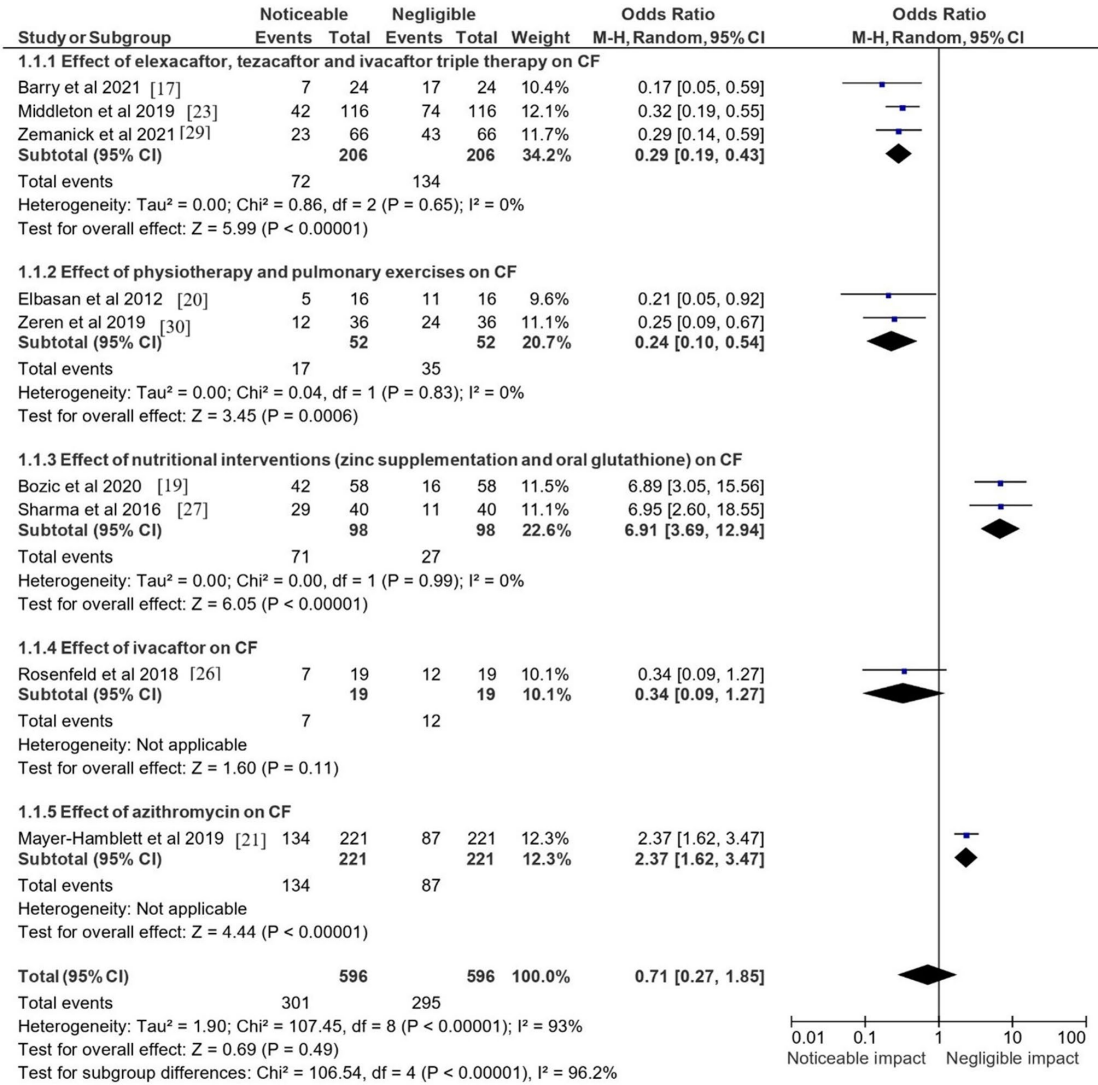
Odds ratio displaying the level of impact/efficacy of the various treatment modalities mentioned in the selected papers. This forest plot presents pooled odds ratios (ORs) with 95% confidence intervals for each treatment modality. Triple therapy and physiotherapy showed significant benefits with low heterogeneity, while nutritional interventions demonstrated non-significant effects. Ivacaftor monotherapy had wide confidence intervals, indicating uncertainty in its efficacy

Figure 4 provides a comprehensive analysis of the included studies investigating the level of impact or efficacy of the various treatment modalities mentioned in the selected papers in terms of their OR. The plot summarized the findings from five categories of CF treatments: triple therapy with elexacaftor, tezacaftor, and ivacaftor; physiotherapy and pulmonary exercises; nutritional interventions (zinc supplementation and oral glutathione); ivacaftor monotherapy; and azithromycin.

Three studies were included for the triple combination therapy: [17, 23, 29]. The pooled odds ratio was 0.29 (95% CI [0.19, 0.43]), indicating a significant benefit of the therapy with a Z value of 5.99 (*p* < 0.00001). The heterogeneity among these studies was low, with an I² of 0%, suggesting that the results were consistent across studies. Physiotherapy and pulmonary exercises were evaluated in two studies [20, 30]. The pooled odds ratio was 0.24 (95% CI [0.10, 0.54]), also showing a significant treatment effect with a Z value of 3.45 (*p* = 0.0006). Similar to triple therapy, no heterogeneity was observed (I² = 0%). The nutritional interventions group included two studies [19, 27], with a stark contrast in odds ratios to the other treatment categories. The nutritional interventions showed an OR of 6.91 (95% CI [3.69, 12.94]), indicating a non-significant effect, with a Z value of 6.05 (*P* < 0.00001). Ivacaftor monotherapy, examined in the study by Rosenfeld et al. [26], had an OR of 0.34 (95% CI [0.09, 1.27]). While the point estimate suggested potential benefit, this result was not statistically significant (*p* = 0.11), and as such, the evidence is less conclusive for this treatment. Regarding azithromycin, Mayer-Hamblett et al. [21] reported an OR of 2.37 (95% CI [1.62, 3.47]), indicating a significant positive effect of the treatment (Z = 4.44, *p* < 0.00001) with no heterogeneity applicable to a single study.

When considering the overall effect across all treatments and studies, the total OR was 0.71 (95% CI [0.27, 1.85]), with a Z value of 0.69 (*p* = 0.49). This overall effect did not reach statistical significance, and substantial heterogeneity (I² = 93%) indicated variability in the effects of different treatments. The subgroup differences were highly significant (Chi² = 106.54, df = 4, *p* < 0.00001, I² = 96.2%), suggesting that the treatment effects varied greatly among the different categories of CF treatments. This emphasizes the need for tailored approaches in CF treatment, as the efficacy of interventions can differ substantially.

**Fig. 5:**
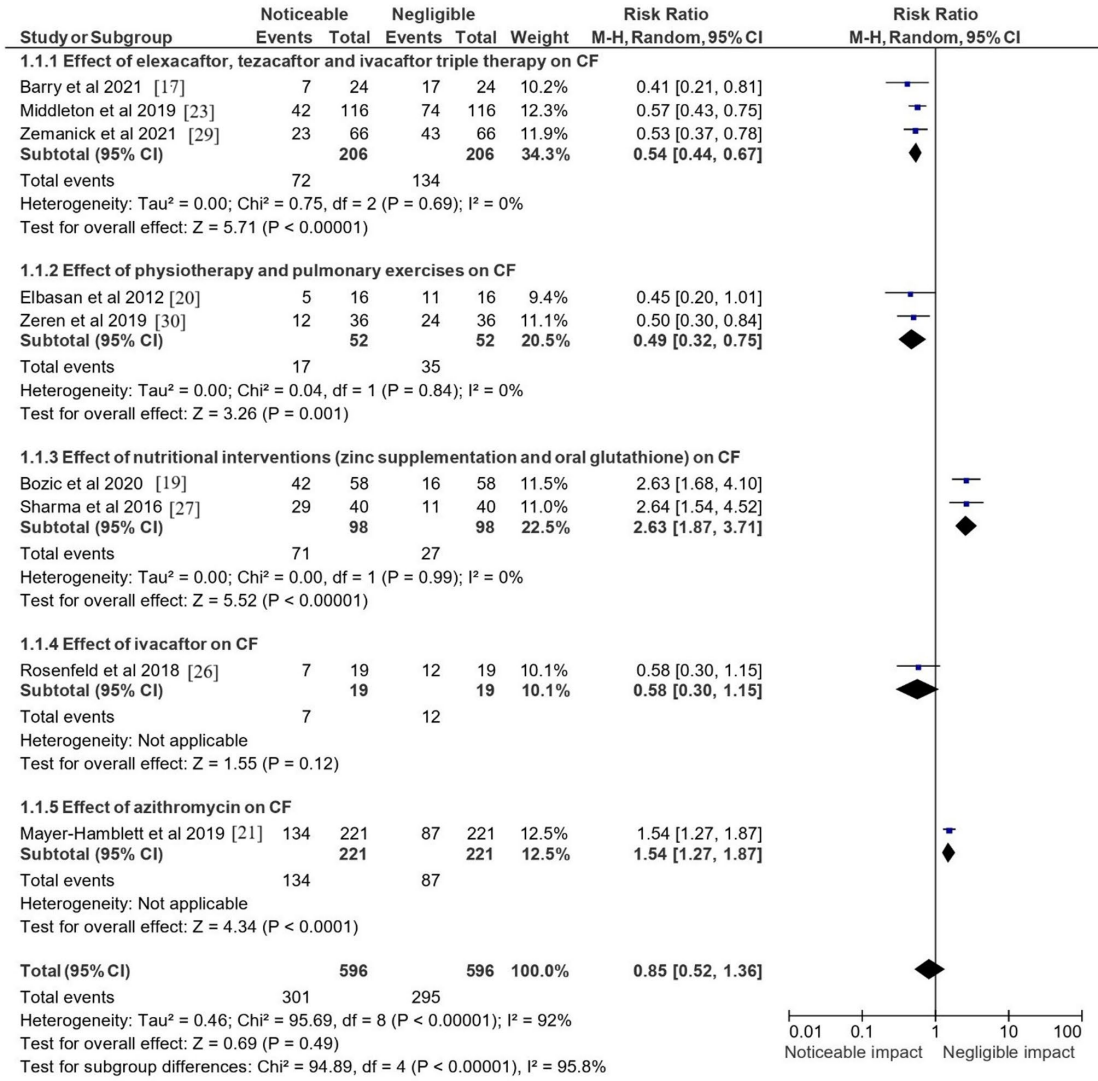
Risk ratio displaying the level of impact/efficacy of the various treatment modalities mentioned in the selected papers. This forest plot displays pooled risk ratios (RRs) with 95% confidence intervals for each intervention. Similar to OR estimates, triple therapy and physiotherapy yielded significant reductions in adverse outcomes, whereas nutritional interventions showed increased risk without clinical benefit. Azithromycin was associated with higher event rates but improved secondary outcomes such as weight gain

A thorough examination of the included papers examining the effectiveness or impact of the different treatment modalities regarding their RR is elucidated in Fig. 5. The categories of elexacaftor, tezacaftor, and ivacaftor triple therapy included three studies: [17, 23, 29]. The individual studies reported RRs of 0.41, 0.57, and 0.53, respectively, all indicating a risk reduction when using the triple therapy compared to the control. The pooled RR for this therapy was 0.54 (95% CI [0.44, 0.67]). The heterogeneity was non-existent (I² = 0%), and the test for overall effect showed a highly significant benefit (Z = 5.71; *p* < 0.00001). The second treatment modality, physiotherapy and pulmonary exercises, was analysed through two studies [20, 30]. The RRs were 0.45 and 0.50, respectively, suggesting a favorable effect of these interventions. The combined RR for physiotherapy and exercises was 0.49 (95% CI [0.32, 0.75]), and no detected heterogeneity (I² = 0%). The overall effect was significant (Z = 3.26; *p* = 0.001). Nutritional interventions, specifically zinc supplementation [19] and oral glutathione [27]showed non-significant effect sizes with RRs of 2.63 and 2.64, respectively. The pooled RR for nutritional interventions was 2.63 (95% CI [1.87, 3.71]). The uniformity of effect sizes across these studies resulted in zero heterogeneity (I² = 0%), and the test for overall effect indicated a significantly increased risk (Z = 5.52; *p* < 0.00001). The effect of ivacaftor monotherapy was assessed in a single study by Rosenfeld et al. [26]which produced an RR of 0.58 (95% CI [0.30, 1.15]). However, this did not reach statistical significance (Z = 1.55; *p* = 0.12), and total events were 7 in the treatment group versus 12 in the control group. The impact of azithromycin was reported by Mayer-Hamblett et al. [21] with an RR of 1.54 (95% CI [1.27, 1.87]), indicating a significant increase in risk associated with the treatment (Z = 4.34; *p* < 0.0001). The total number of events was 134 in the treatment group compared to 87 in the control group.

The combined RR across all treatments and studies was 0.85 (95% CI [0.52, 1.36]). However, this overall estimate did not achieve statistical significance (Z = 0.69; *p* = 0.49), and there was substantial heterogeneity (I² = 92%), suggesting considerable variation in treatment effects. The test for subgroup differences was highly significant (Chi² = 94.89, df = 4; *p* < 0.00001, I² = 95.8%), reinforcing the variability and indicating that the effects of CF treatments varied significantly across different categories.

## Discussion

The study’s results are significant because they provide valuable insights into the efficacy and safety of different treatment modalities available for children with cystic fibrosis. The analysis identified the most effective treatment modalities while also highlighting potential areas for future research. The study’s findings can inform clinical decision-making, guide the development of treatment guidelines, and improve patient outcomes.

Alton et al. [16] provided evidence for the modest efficacy of gene therapy in CF, with the pGM169/GL67A complex showing a stabilization effect on lung function without increasing adverse events. This finding was paralleled by the substantial improvements in lung function and QoL reported by Barry et al. [17] and Middleton et al. [23]who both evaluated the *CFTR* modulator combination of elexacaftor–tezacaftor–ivacaftor, albeit in different patient genotypes. Their results not only reinforced the therapeutic potential of *CFTR* modulators but also highlighted an acceptable safety profile. Contrastingly, Bozic et al. [19] reported no significant benefit of oral glutathione in improving growth or inflammatory markers, which diverged from the overall trend of positive outcomes seen in other studies. This underscores the variability in response to different therapeutic agents within the CF population.

The benefits of non-pharmacological interventions were evident in the work of Elbasan et al. [20]who underscored the importance of physical therapy, and Boon et al. [18]who illustrated the utility of digital health interventions for self-management, especially in pediatric patients. Similarly, Mayer-Hamblett et al. [21] identified an adjunctive benefit of azithromycin in reducing pulmonary exacerbations, suggesting a multifaceted approach to therapy.

The lack of protective effect against aminoglycoside-induced nephrotoxicity by rosuvastatin, as reported by McWilliam et al. [22]left the field with an inconclusive hypothesis, reflecting the complexity of addressing CF comorbidities. Modi et al. [24] showed that adherence to treatment regimens was problematic, with objective measures indicating low adherence rates. This finding presents a challenge in clinical practice and highlights the discrepancy between self-reported and actual adherence. Ratjen et al. [25] contributed to the evidence supporting the safety and efficacy of inhaled hypertonic saline for improving lung ventilation, consistent with the overall positive findings related to pulmonary treatments. Interventions aimed at preserving pancreatic function in the very young were positively reported by Rosenfeld et al. [26]suggesting that early treatment initiation could be beneficial, which was a recurring theme of improving outcomes through early intervention.

However, Sharma et al. [27] and Voldby et al. [28] found limited benefits from zinc supplementation and LCI-triggered bronchoalveolar lavage, indicating that not all interventions yield significant clinical improvements and underscoring the need for careful treatment selection. Zemanick et al. [29] and Zeren et al. [30] provided insights into the effectiveness of chest physiotherapy and the nuanced benefits of additional inspiratory muscle training, specifically improving maximal inspiratory pressure.

The lungs are in direct contact with the outside world along with other organs, including the skin and intestines [31]– [32]. Systemic drug administration through inhalation (oral and nasal) is an efficient substitute for parenteral drug delivery to treat pulmonary disease. There are several advantages to administering drugs through the lungs, including greater patient compliance, a quick beginning of action, non-invasiveness, high lung permeability [33]and rapid absorption [34]. As a result, this method has been employed for the local administration of many different types of medications, including antibiotics, chemotherapeutics, peptides, and vaccinations [35]. The most popular antimicrobials for CF are now all available in inhalation formulations, as detailed below. These include tobramycin, colistimethate sodium, and aztreonam lysine [36]– [37].

Antibiotics and painkillers are currently frequently used to treat the symptoms of infections and inflammations linked to CF. Bronchodilators, mucolytics, and osmotic agents are also given [38] to enhance airway and sputum clearance. In addition to the treatment modalities evaluated in our meta-analysis, it is important to consider the role of mucolytic agents such as dornase alfa in the management of CF. Dornase alfa, a recombinant human deoxyribonuclease (DNase), plays a key role in reducing sputum viscosity by cleaving extracellular DNA released from neutrophils in the airways — a hallmark of chronic pulmonary inflammation in CF patients [38].

Chronic airway inflammation, which is a common complaint among CF patients, eventually triggers several physiological and metabolic abnormalities, such as anorexia, weight loss, and metabolic collapse. Anti-inflammatory therapy is, therefore, a different choice from antibacterial therapies in preventing a deterioration in lung function [31]. In multiple investigations, anti-inflammatory medications have been demonstrated to be helpful, but their usage as CF treatments has been constrained due to the associated side effects [39–41]. Systemic corticosteroids and high-dose ibuprofen were the first anti-inflammatory medications tested in CF [42–46].

In the review presented by Wilson et al. [47]the focus was on evaluating the effectiveness and safety of various airway clearance techniques in individuals with cystic fibrosis. While the reviews were considered to have a low risk of bias, the underlying trials often lacked sufficient information to assess bias thoroughly, particularly in reporting outcomes, which introduced a high risk of reporting bias. A moderate quality of evidence suggested no significant difference in FEV1 between positive expiratory pressure (PEP) therapy and oscillating devices after six months. However, the evidence quality needed to be higher or, for most other outcomes and comparisons, higher, preventing definitive conclusions. On the other hand, the systematic review by Mielus et al. [48] explored the gastrointestinal manifestations of CF, with a particular emphasis on nutritional interventions and their effects on anthropometric measures. Their review highlighted the benefits of individualized nutritional plans, a high-fat, high-calorie diet, and the use of enteric tube feeding based on observational data.

In contrast, no significant impact on anthropometric measures was observed by supplementing probiotics, lipids, docosahexaenoic, glutathione, or antioxidant-enriched multivitamins. Comparing the outcomes of our review with the findings of Wilson et al. [47] and Mielus et al. [48] reveals both similarities and divergences. Like Wilson et al., our review likely dealt with the complexities of treatment efficacy and the challenges in drawing clear conclusions due to variability in study design, insufficient reporting, and potential biases. Both reviews underscore the importance of high-quality evidence and careful interpretation of results.

The review by Schneider et al. [49] focused on the advancements in *CFTR* modulator therapies, gene therapy, and the potential of CRISPR/Cas9 gene editing as treatments for cystic fibrosis. They highlighted the significant progress made with the introduction of ivacaftor and its combination with lumacaftor or tezacaftor in treating most CF patients. Despite the early promise of gene therapy, the review acknowledged the challenges faced in effectively delivering *CFTR* genes to the lungs, indicating that while gene therapy was a hopeful avenue, practical applications had been less successful than anticipated. They also pointed to the future potential of these technologies in CF treatment. The findings of Rafeeq et al. [46] provided an extensive overview of the current drug therapies for CF and explored future therapeutic developments. They emphasized the complexity of CF as a multisystem disorder primarily affecting the respiratory and gastrointestinal systems. Similar to Schneider et al., they mentioned the emerging role of protein rectifiers or corrector drugs, which aim to address the underlying structural and functional abnormalities associated with *CFTR* variants. They also recognized the potential of gene therapy and the need for targeting cellular interactomes, as well as the development of new drugs for symptomatic relief.

Comparing these reviews to our findings, several similarities and differences become evident. Schneider et al. [49] and Rafeeq et al. [46]as well as our review, likely emphasized the transformative impact of *CFTR* modulators on the treatment landscape of CF, which represents a significant shift from symptomatic treatment to targeting the underlying cause of the disease. The challenges and limited success with gene therapy delivery methods may have been a point of concordance among the reviews, highlighting an area where high expectations still need to translate into clinical success. While potentially broader in scope, our review shares the optimism about the future of CF treatment expressed by Schneider et al. [49] and Rafeeq et al. [46]particularly regarding novel therapeutic approaches like CRISPR/Cas9 and other gene-editing technologies. Additionally, our review might have discussed the importance of early intervention and the role of physical therapy and digital health interventions, which should have been explicitly covered by Schneider et al. [49] or Rafeeq et al. [46].

Lee et al. [50] acknowledged that while new small molecule modulators have significantly advanced treatment for certain CF variants, these therapies are only sometimes effective, with rare *CFTR* variants remaining particularly challenging to treat. Their review highlighted the promise of new precise gene targeting methods that might lead to effective personalized therapies, potentially replacing or complementing classical approaches that target specific disease-causing variants. Our review differed in emphasis, covering a wider range of therapeutic approaches beyond gene editing, including the latest developments in *CFTR* modulator therapy, advancements in symptomatic treatments, and improvements in care standards that contribute to the increased life expectancy of CF patients. Lee et al. [50] further pointed to a significant increase in the mean survival age of CF patients in Canada, a testament to the overall progress in CF care. Our review might have also cited similar statistics or trends, indicating improvements in patient outcomes due to advancements in treatment. A potential similarity between our review and that of Lee et al. [50] could be the shared optimism about the future of CF treatments due to rapid advancements in gene editing technologies. Both reviews likely highlighted the promise of these technologies in developing mutation-agnostic therapies that could benefit a wider range of CF patients.

### Limitations

There are caveats to the current research that must be noted. First, some relevant studies were probably likely overlooked despite a thorough search of internet databases using acceptable terms for searching, bibliographic databases, and sources. The breadth of the investigation was likely constrained since only English-language articles were included. Second, the included studies varied in sample size, with some having a relatively small number of patients, which may limit the generalizability of the findings. Additionally, the heterogeneity was noted across treatment modalities, mainly due to the differences in study design, patients’ clinical features, outcome definitions, and intervention methods. Although subgroup analyses were done to decrease these variabilities, residual heterogeneity remains a concern, especially when interpreting pooled estimates across diverse interventions. Notably, while RCTs were generally assessed as having low to moderate risk of bias, observational studies introduced potential confounding factors that may influence effect estimates. Furthermore, geographic representation was largely limited to developed countries, which may limit the applicability of findings in resource-limited settings where access to advanced therapies such as CFTR modulators is restricted. Moreover, the review primarily focused on the analysis of *CFTR* modulators, complementary therapeutic strategies, exercise, and gene therapy, potentially overlooking other treatment modalities that could impact the management of CF in children. Finally, the review highlighted the need for more clinical trials, indicating the limited availability of robust evidence in certain areas. Therefore, the findings and conclusions ought to be evaluated cautiously, and more study is required to establish more definitive therapeutic strategies for children with CF, considering a more holistic approach to the disease rather than solely addressing symptoms.

### Recommendations about the clinical domain

Several recommendations can be made for managing CF using the findings obtained from this review. Firstly, it is recommended that triple therapy (elexacaftor/tezacaftor/ivacaftor) be considered as a potential treatment to stabilize lung function in CF patients, particularly those with Phe508del-gating or residual function genotypes. The use of *CFTR* modulators should be strongly considered where appropriate, as they have demonstrated significant improvements in lung function, QoL, and reduction in sweat chloride concentrations, with an acceptable safety profile. Digital health tools such as mobile applications for self-management of pancreatic enzyme replacement therapy may also support adherence and improve GI QoL, particularly in older children and adolescents. Physical therapy, including chest physiotherapy and aerobic exercise training, is recommended to improve physical fitness and muscle endurance, which are important components of comprehensive CF care.

The addition of azithromycin to antimicrobial regimens for children with early *Pseudomonas aeruginosa* infection should be considered due to its potential to reduce pulmonary exacerbations and promote weight gain. However, caution should be exercised with interventions that have not demonstrated clear benefits, such as oral glutathione supplementation and certain supplements like zinc, which may not yield significant improvements in growth, inflammatory markers, or the need for antibiotics. Treatment adherence is critical for achieving the best outcomes in CF care. Therefore, it is recommended that adherence assessments be actively monitored and supported, utilizing both self-reported and objective measures to address discrepancies and improve the accuracy of adherence assessments.

Early intervention strategies, particularly for very young children with CF, are advised to preserve pancreatic function and potentially improve long-term outcomes. Given their safety and effectiveness, non-pharmacological treatments, such as inhaled hypertonic saline, should be considered for improving lung ventilation inhomogeneity. Moreover, while comprehensive chest physiotherapy is recommended for improving various physical health parameters, adding inspiratory muscle training may be reserved for targeted therapy, as it has shown specific benefits in increasing maximal inspiratory pressure.

## Conclusion

It was found that *CFTR* such as elexacaftor, tezacaftor, and ivacaftor helped to lessen bacterial infections and other illnesses linked to CF to some extent. Additionally, it was discovered that regular exercise and complementary therapeutic strategies had a beneficial impact on the children’s general QoL. Gene therapy was seen to have a considerable effect, but more research and trials are needed. None of the other treatment techniques indicated in the other investigations showed any additional noteworthy differences. The literature search yielded several articles that mentioned gene therapy and nanotechnology, but they needed more supporting data to justify further analysis. Scoping and literature reviews constituted a disproportionately high proportion of the literature on innovative methods. So, more clinical trials are required to determine specific treatment methods that focus on a more holistic rather than symptomatic approach to this condition.

## Supplementary Information

Supplementary Material 1.

## Data Availability

Availability of data and materials: This systematic review and meta-analysis article includes all data generated or analyzed during this study.
